# Testicular fat deposition attenuates reproductive performance via decreased follicle-stimulating hormone level and sperm meiosis and testosterone synthesis in mouse

**DOI:** 10.5713/ab.23.0175

**Published:** 2023-08-28

**Authors:** Miao Du, Shikun Chen, Yang Chen, Xinxu Yuan, Huansheng Dong

**Affiliations:** 1 College of Animal Science and Technology, Qingdao Agricultural University, Qingdao 266109, China; 2 College of Veterinary Medicine, Murdoch University, Murdoch, Western Australia 6150, Australia; 3 Department of Pharmacology and Toxicology, School of Medicine, Virginia Commonwealth University, Richmond, VA 23284, USA

## Abstract

**Objective:**

Testicular fat deposition has been reported to affect animal reproduction. However, the underlying mechanism remains poorly understood. The present study explored whether sperm meiosis and testosterone synthesis contribute to mouse testicular fat deposition-induced reproductive performance.

**Methods:**

High fat diet (HFD)-induced obesity CD1 mice (DIO) were used as a testicular fat deposition model. The serum hormone test was performed by agent kit. The quality of sperm was assessed using a Sperm Class Analyzer. Testicular tissue morphology was analyzed by histochemical methods. The expression of spermatocyte marker molecules was monitored by an immuno-fluorescence microscope during meiosis. Analysis of the synthesis of testosterone was performed by real-time polymerase chain reaction and reagent kit.

**Results:**

It was found that there was a significant increase in body weight among DIO mice, however, the food intake showed no difference compared to control mice fed a normal diet (CTR). The number of offspring in DIO mice decreased, but there was no significant difference from the CTR group. The levels of follicle-stimulating hormone were lower in DIO mice and their luteinizing hormone levels were similar. The results showed a remarkable decrease in sperm density and motility among DIO mice. We also found that fat accumulation affected the meiosis process, mainly reflected in the cross-exchange of homologous chromosomes. In addition, overweight increased fat deposition in the testis and reduced the expression of testosterone synthesis-related enzymes, thereby affecting the synthesis and secretion of testosterone by testicular Leydig cells.

**Conclusion:**

Fat accumulation in the testes causes testicular cell dysfunction, which affects testosterone hormone synthesis and ultimately affects sperm formation.

**Keywords:**

Cholesterol Metabolism, Overweight, Spermatogenesis, Testosterone

## INTRODUCTION

Obesity is a prevalent issue worldwide and is a significant public health concern [1,2]. It has been demonstrated that obesity can have adverse effects on mammalian reproductive capacity [1,3,4]. To the reproduction related hormonal secretion, obesity hindered testosterone production, impeded sexual development, and reduced male reproductive capability by disrupting the testicular leptin transduction action pathway [5]. There are also relevant studies shows that obesity in adult male rats will cause the spermatogonia and S-phase population in meiosis to be increased [6]. While numerous studies examine the correlation between overall fat accumulation and reproductive ability in animals, however, little research has been conducted on the specific effects of testicular fat accumulation. Given the current trend of over-nutrition, researchers are focusing on the correlation between obesity and male fertility.

Cholesterol plays a crucial role in the synthesis of testosterone in testicular Leydig cells [7]. Obesity has been linked to decreased testosterone production [8]. Obesity also causes a significant increase in the body’s pool of cholesterol, which can range from 25% to 50% in adipose depots [9]. Studies have also shown a positive correlation between cholesterol levels and the development of obesity [10]. In addition, changes in the esterified cholesterol in testicular seminiferous tubules have been observed in individuals with arrested spermatogenesis [11]. Therefore, it is crucial to further investigate the molecular mechanisms and regulatory pathways of obesity that affect spermatogenesis.

Increasing evidence suggests that semen quality can be affected by diet, and diet-induced overweight male mice show the decreased sperm motility [12]. Previous studies have shown that obesity affects endocrine regulation of male reproductive function and fertility, especially spermatogenesis [13–15]. Mammalian spermatogenesis is a multi-step process and is regulated by a variety of endocrine hormones, including follicle-stimulating hormone (FSH) and luteinizing hormone (LH) secreted by the pituitary gland. After numerous mitotic divisions, a proportion of the spermatogonia enters meiosis and develop into spermatocytes that eventually give rise to sperms with fertilization ability in the epididymis [16,17]. Meiosis of the spermatogenesis is divided into meiosis I and meiosis II and the meiosis I is further divided into five stages, including leptotene, zygotene, pachytene, diplotene, which are allow exchange of genetic material between homologous chromosomes of primary spermatocytes and the production of haploid spermatids [18,19]. Spermatogenesis is also regulated by testosterone. Studies have shown that the conversion of round spermatids between stages VII and VIII is a highly testosterone-dependent step during spermiogenesis [20]. So, it is important to study the effects of testicular fat deposition on the levels of FSH and LH, sperm meiosis and testosterone synthesis.

In this regard, a high-fat diet (HFD) was used to induce obesity in CD1 mice, which were compared to control mice on a normal diet (CTR). Using a sperm analyzer and immunohistochemistry (IHC) staining, we confirmed the effects of obesity on sperm quality and spermatogenesis. Subsequently, we examined whether these effects were associated with sperm meiosis and testosterone synthesis. Our findings suggest that fat accumulation in the testes leads to dysfunction of testicular cells, which affects the synthesis of the testosterone hormone and ultimately affects the formation of sperm.

## MATERIALS AND METHODS

### Mouse model

The model was constructed by randomly dividing 4-week-old CD1 male mice into two groups, each containing 20 mice. All mice were purchased from Charles River, strain code 201, strain name CD-1 (ICR) IGS. The adaptation period for mice is one week. Illumination was provided between 7:00 am to 7:00 pm every day. The ambient temperature and relative humidity were maintained at 22°C±2°C and 55%±5%, respectively. These standards were maintained in specific pathogen-free (SPF) conditions. One group was fed a HFD from Qingdao Baisaisi Biotechnology Co. (Qingdao, China; Table 1), while the other was fed a normal diet (Shuyushengwu, SYNCM) with Ain93M standard for 10 weeks. The mice fed the HFD were labeled as DIO (diet-induced overweight), while those fed the normal diet were labeled CTR. At the end of each experiment, mice were anesthetized, and blood was collected by retroorbital bleeding into a heparinized tube. After an animal was euthanized, testes, epididymides, and vas deferens were removed and placed into a 30-mm sterile plastic dish. One testicle was retained for subsequent experiments, the other testicle and tissue was cut five times in the normal saline at 37°C, to allow sperm to move out from the tissue for 10 min. All procedures described in this study were reviewed and approved by the Ethics Committee of Qingdao Agricultural University (Approval number 2020 0514).

### Sperm motility analysis

The WHO motility parameters (PR+NP, total motility; PR, progressive motility) were assessed by a sperm class analyzer (SCA, Microptic S.L., Barcelona, Spain). Integrated hardware components included a phase contrast microscope with stroboscopic illumination, a camera, a warm stage, an image digitizer, and a computer to analyze and save the data. Semen aliquots (4 μL) from DIO and CTR group were placed in a Leja 4 analysis chamber (Leja Products B.V, Nieuw-Vennep, The Netherlands). Six randomly selected microscopic fields were scanned. After every scan, the playback showed the video sequences to determine whether all spermatozoa were identified and to reconstruct their trajectory by SCA system.

### Tissue section production

Testes and caudal epididymis were dissected immediately by the mechanical separation method. Then, the tissues were fixed in Bouin’s fixative (Solarbio, Beijing, China) overnight at 4°C, dehydrated in graded ethanol, and embedded in paraffin. Next, 5-μm sections were cut with a microtome, followed by deparaffinization and rehydration, then stained with hematoxylin and eosin (Beijing Kyushu Berlin Biotechnology Co., Beijing, China) for histological analysis. The images were acquired using the Leica DM750 biologic microscope.

### Oil red staining

First, the frozen sections of testicular tissue were prepared. Second, the optimum cutting temperature compound was placed on the sections. After fixation with 10% formaldehyde for 10 min, the sections were washed with phosphate-buffered saline (PBS) and subjected to 60% isopropyl alcohol pre-dyeing and oil red staining. Finally, the nucleus was stained by hematoxylin and the sections sealed with glycerol for storage until they could be examined.

### Immunohistochemistry

Paraffin sections were placed into PBS after dewaxing, rehydration, and staining. Then, the sections were boiled for 15 min in sodium citrate buffer for antigen retrieval. Subsequently, after the sections were blocked with 5% bovine serum albumin (BSA; Roche, Basel, Switzerland), probed with a primary antibody (Anti-VASA/VAS; Abcam, Cambridge, UK), diluted 1:200 in a blocking solution, at 4°C overnight and washed three times in PBS. Then, the sections were treated with 3% H_2_O_2_ to eliminate the internal peroxidase activity and incubated with horseradish peroxidase-conjugated immunoglobulin G (IgG) (Beyotime, Shanghai, China), diluted 1:150 in PBS, for 90 min at 37°C. The list of antibodies is shown in Table 2. Finally, the sections were stained with 3,3-diaminobenzidine, and the nuclei were stained with hematoxylin. The images were captured using laser scanning confocal microscope.

### Spermatocyte surface spreading

The testes were dissected at room temperature. The renal tubules were rinsed with PBS at pH = 7.4 and placed in hypotonic extraction buffer (30 mM Tris, 50 mM sucrose, 17 mM trisodium citrate dihydrate, 5 mM ethylenediaminetetraacetic acid (EDTA), 0.5 mM DTT and 0.5 mM phenylmethylsulfonyl fluoride, pH = 8.2) for 30 to 60 min. Subsequently, the tubules were opened on a clean glass slide containing 100 mM sucrose (pH 8.2) and repeatedly blown to make a suspension. The cell suspension was placed on a glass slide containing 1% paraformaldehyde and 0.15% Triton X-100 at pH 9.2. The slides were dried in a high humidity closed box for at least 2 h, washed twice with 0.4% Photoo (Kodak, 1464510, Rochester, NY, USA), and dried at room temperature.

### Immunofluorescence

The spermatocytes for different periods were fixed on the slide and washed two times with PBS containing 0.1% Triton X-100, each for 10 min. After blocking with 5% BSA (Roche, Switzerland), each section was incubated with primary antibody Anti-MutL protein homolog 1 (MLH1), BD Pharmingen TM [21]; Anti-VASA, anti-Synaptonemal complex protein 3 (SYCP3), Abcam (UK) [22]; Anti-H2A histone family member X S139 (γH2AX), Millipore [23]; Anti-synaptonemal complex protein 1 (SYCP1), Novus Biologicals [24], both diluted 1:200 in a blocking solution, at 4°C overnight and washed in PBS three times. Then, the sections were incubated with goat FITC-conjugated secondary antibody to rabbit and TRITC-conjugated secondary antibody to mouse (1:200) for 1 h at 37°C. Next, the nuclei were stained with 4,6-diamidino-2-phenylindole (DAPI), and images were acquired using a microscope purchased from Leica Microsystems (Buffalo Grove, IL USA). The list of antibodies is shown in Table 3.

### Serum hormone test

The mice were placed in a suitable restraint with towel wrap, to expose the tail or gently immobilize it. A 1 mL syringe with a fine-gauge needle was inserted into the tail vein to withdraw blood. The collected blood was placed in centrifuge tubes and spun at 2000 RPM for about 10 minutes. After centrifugation, the serum was collected from the top layer. FSH and LH levels were measured by ELISA (FSH ELISA Kit, FineTest, EM1035; LH ELISA Kit, FineTest, EM1188; Wuhan Fine Biotech Co., Ltd., Wuhan, China).

### Testing the content of testosterone and extracting cholesterol

One testicle from CTR and DIO mice was ground separately and weighed. The fresh material was homogenized in a porcelain beaker containing 0.01 M phosphate buffer using an Ultra-Turrax homogenizer. The homogenate was then placed in a shaker for 2 h and centrifuged at 4,500 rpm for 15 min. The supernatant was transferred to a clean tube and an organic solvent (diethyl ether) was added to the tube and the testosterone extracted from the aqueous phase. The samples were placed in standard and blank wells, with the sample in triplicate. Testosterone levels were measured by ELISA (Testosterone ELISA Kit, FineTest, EU0400). Total cholesterol, free cholesterol, high-density lipoprotein cholesterol (HDL) and Low-density lipoprotein cholesterol (LDL) in each sample were measured using a specific reagent kit (Cholesterol Assay Kit; Abcam, UK; AB65359) (Cholesterol Assay Kit - HDL and LDL/VLDL, AB65390).

### Real-time polymerase chain reaction

Total RNA was extracted from sperm of experimental mice using a Trizol Kit (Life Technologies, Carlsbad, CA, USA). cDNA was synthesized using the PrimeScript RT Reagent Kit (TaKaRa, RR037A, Kusatsu, Japan). Real-time polymerase chain reaction (PCR) was performed with a Roche Light Cycler 480II System. SYBR Premix Ex TaqTM kit (TaKaRa Corporation, Japan) were used in the experiment. The primers used to amplify are listed in Table 3. Reaction system: cDNA, 1 μL; SYBR Premix Ex TaqTM (2×), 5 μL; RNase-free water, 3.6 μL; Forward primer, 0.2 μL (5 μM); Reverse primer, 0.2 μL (5 μM); a total of 10 μL. Reaction conditions were as follows: 40 cycles of 95°C for 10 min, 95°C for 15 seconds and 60°C for 1 min. The relative expression level of mRNAs was normalized to GAPDH internal reference gene. All qRT-PCR runs were repeated thrice. The results were calculated using 2^−ΔΔCt^ method.

### Statistical analysis

The experiments had 20 mice each in the control and 20 in the experimental group. The sperm analysis used five mice from each group. Ten mice from each group were used to make testicular tissue sections for hematoxylin-eosin (HE) staining, oil red staining, and immunohistochemistry. Five mice were used for immunofluorescence in each group. Significant differences among experimental groups were determined by one-way analysis of variance followed by Tukey’s multiple comparison test, and unpaired t-test was used for the binomial data. A p-value <0.05 was considered significant, a p-value <0.01 was considered extremely significant difference. Data are presented as the mean±the standard error.

## RESULTS

### Effects of DIO mice on mouse body weight, food intake, the numbers of offspring and serum hormone levels

**Figure 1:**
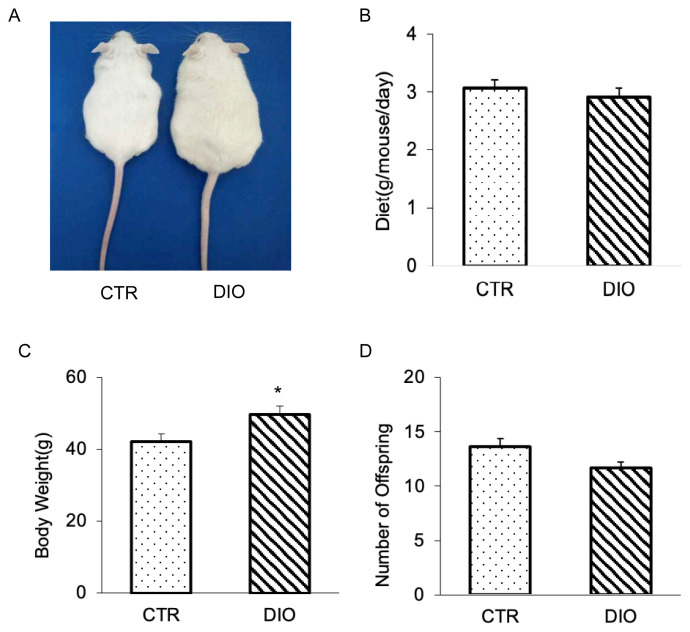
The result of constructing overweight mouse. (A) Photographs of mice at age 15 weeks; they consumed either high-fat or control diet since the age of 4 weeks. (B) Intake difference in mice consuming the high fat vs control diet at age 15 weeks. (C) Body weight analysis of 20 mice for each group. (D) Comparison of the number of offspring in control and overweight mice. * p<0.05.

To investigate the potential role of fat deposition during spermatogenesis, we constructed a mouse model. Our findings revealed that DIO mice exhibited a significantly larger body shape and weight compared to CTR mice (Figure 1A and 1C), despite being no significant difference in free food intake of food and water between the two groups (Figure 1B). Interestingly, we also observed that the number of offspring produced by the DIO mice decreased, but there was no difference from CTR group (Figure 1D). Compared with the CTR group, DIO mice have significantly lower serum FSH levels (p<0.05). The average serum LH levels of DIO mice was lower than the CTR group, but it showed no significant difference (p>0.05). These results suggest that in mice overweight may impact the spermatogenesis process.

### DIO mice decreased sperm quality

**Figure 2:**
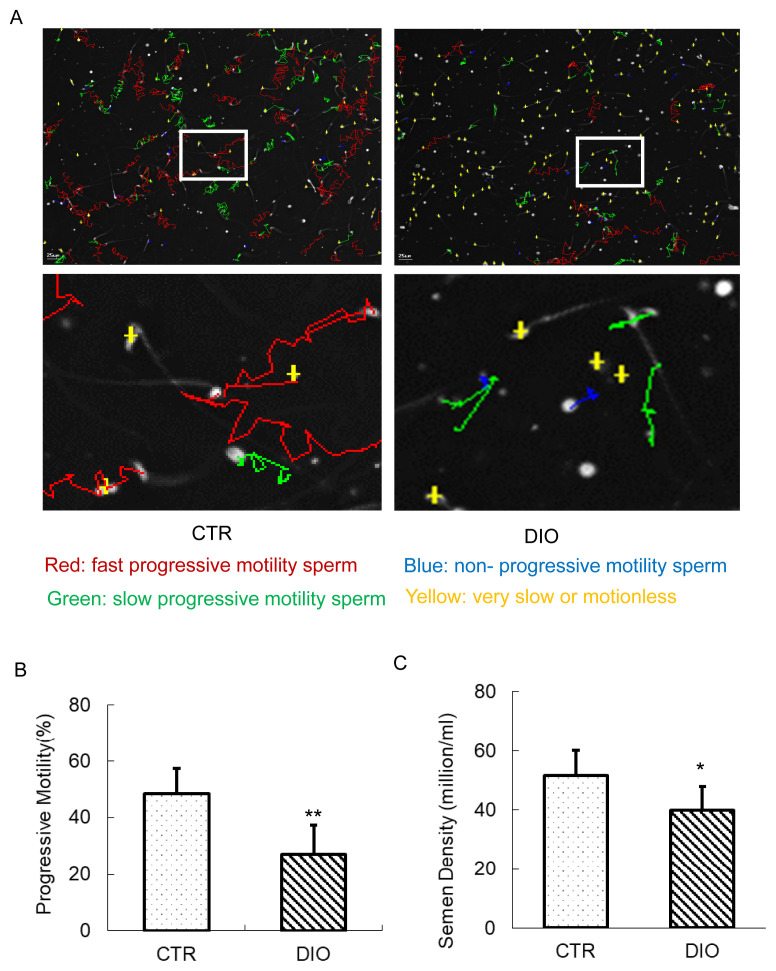
The estimation of sperm motility and semen density. (A) Mouse sperm motility in the control group and in the DIO group. Different colors describe the different states of motions of the sperm. (B) The comparison of progressive motility in the control and DIO mice. (C) Semen density analysis of control and DIO mice. DIO, diet-induced overweight. * p<0.05.

The results of the sperm analyzer showed the movement of sperm in both the CTR and DIO groups (Figure 2A). The analysis revealed that the percentage of average forward movement of sperm in the DIO group was significantly lower than in the CTR group (p<0.01) (Figure 2B). Additionally, we observed that the semen density of the DIO group was lower than that of the CTR group (p<0.05) (Figure 2C). These findings suggest that fat accumulation in mice has a significant impact on both sperm count and quality.

### DIO mice inhibit spermatogenesis

**Figure 3:**
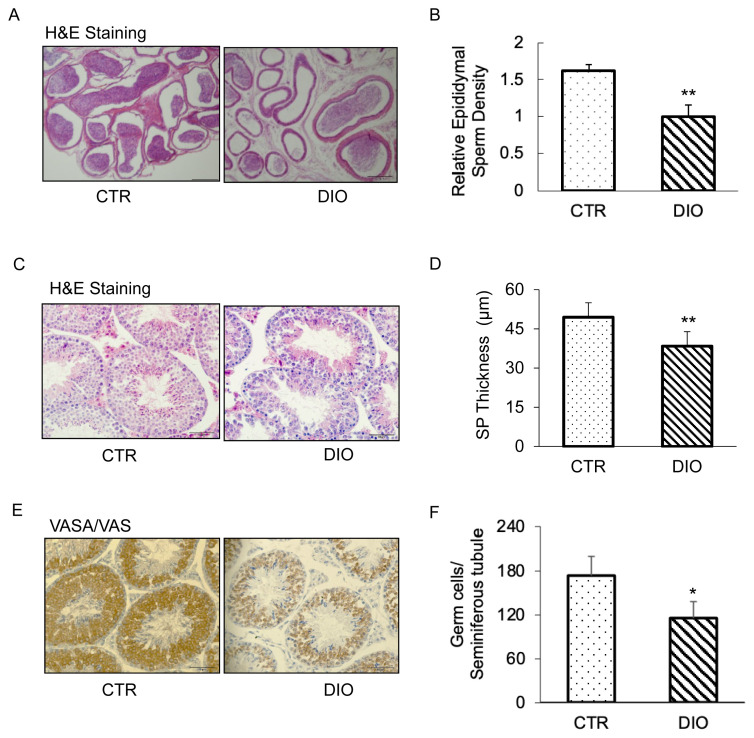
Histological analysis of the seminiferous tubules and caudal epididymis and immunohistochemistry (IHC) analysis of the control and overweight mice. (A) Histological analysis of the caudal epididymis of the control and overweight mice. Scale bars, 200 μm. The control and overweight mice were 15-weeks-old. (B) Relative epididymal sperm Density of the two groups. (C) Histological analysis of the seminiferous tubules of the control and overweight mice. Scale bars, 100 μm. (D) The thickness of the seminiferous epithelium of the two groups. (E) VASA IHC analysis of the germ cells in control and overweight mice depicted the number of germ cells. (F) Quantification of germ cells in the seminiferous tubules of the two groups. * p<0.05.

To investigate the effects of fat deposition on germ cells, we conducted histological examination and IHC analysis of the testis. The results of HE staining showed that the number of mature sperms in the epididymis of the DIO group was significantly lower than that in the CTR group (Figure 3A and 3B), and the testicular cells in the DIO group were less tightly arranged and less round compared to those in the CTR group (Figure 3C). Moreover, statistical analysis of the thickness of the testicular seminiferous tubules revealed an extremely significant difference between the DIO and CTR groups (p<0.01) (Figure 3D). Additionally, the results of immunohistochemistry using VASA/VAS protein as a conserved germline marker, along with statistical analysis, revealed a significant reduction in the number of germ cells in the testes of the DIO group compared to the CTR group (p<0.05) (Figure 3E and 3F). Based on these findings, we hypothesize that fat deposition may inhibit spermatogenesis, thereby leading to a reduction in sperm count.

### The effect of fat deposition on meiosis

**Figure 4:**
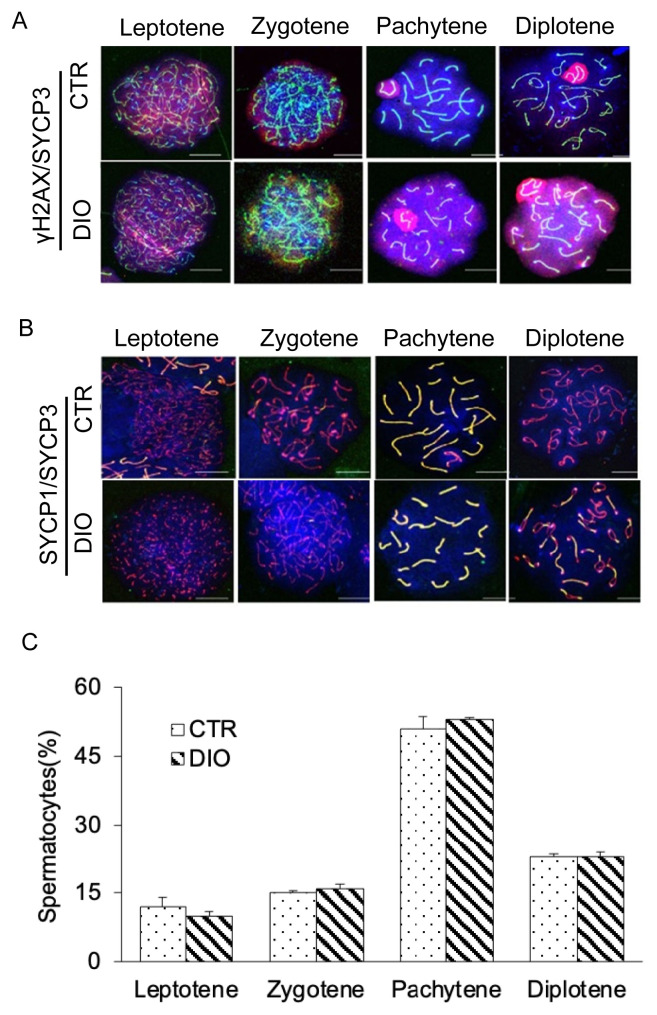
Observation of four periods of meiosis of spermatocytes. (A) Spermatocytes were stained for γH2AX (red) and SYCP3 (green) in the control group and DIO group. Scale bars, 10 μm. (B) Spermatocytes were stained for SYCP3 (red), SYCP1(green), and DAPI (blue) in the control group and DIO group. Scale bars, 10 μm. (C) Statistics of spermatocytes in DIO group and control group at each stage of meiosis. γH2AX, H2A histone family member X; SYCP3, synaptonemal complex protein 3; DIO, diet-induced overweight; DAPI, 4′-6-diamidino-2-phenylindole.

**Figure 5:**
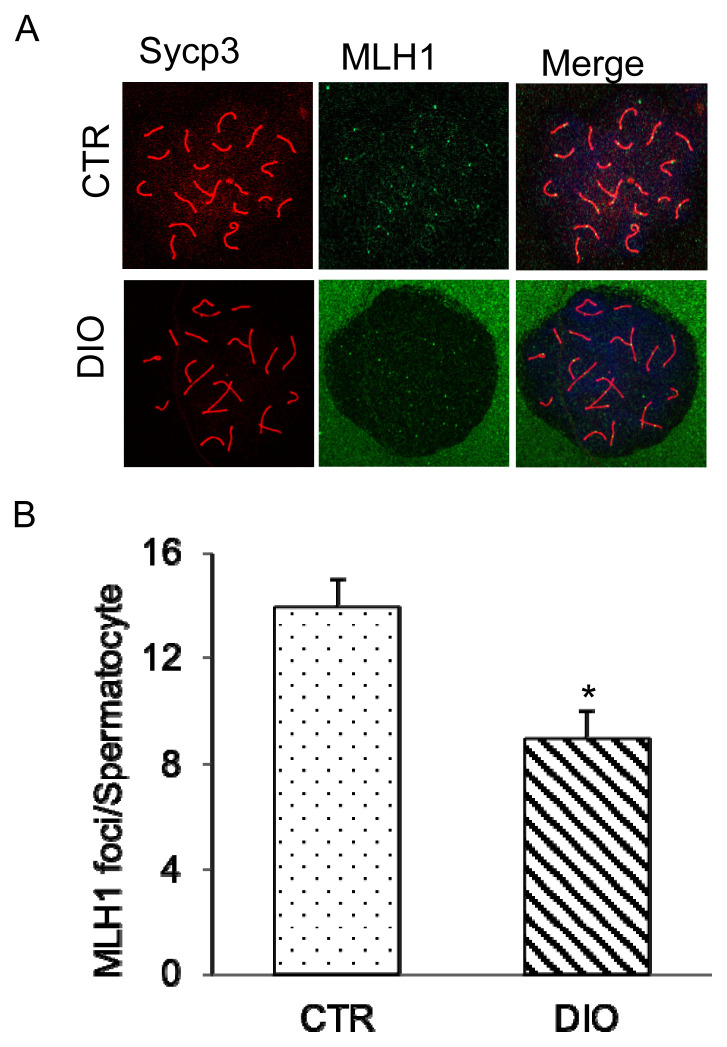
Analysis of expression of MLH1 during the DNA mismatch repair in sperm meiosis. (A) Control and overweight mice spermatocytes were stained for SYCP3 (red) and MLH1 (green). Scale bars, 10 μm. (B) The location of mouse spermatocyte MLH1 and the crossover site statistics. MLH1, MutL homolog 1; SYCP3, synaptonemal complex protein 3. * p<0.05.

Fluorescence monitoring was used to examine important marker molecules for meiosis, including Leptotene, Zygotene, Pachytene, and Diplotene. The results showed no significant difference in the process of chromosome crossover during meiosis between the DIO and CTR groups as shown in Figure 4A and 4B. Moreover, there was no difference in the number of spermatocytes at each stage of meiosis between the two groups (Figure 4C). However, fluorescence monitoring of MLH1 revealed a significant difference in the number of chromosomal crossover sites between the DIO and CTR groups (p<0.05) (Figure 5A and 5B). These results suggest that fat deposition affects the process of meiosis, particularly in the cross-exchange of homologous chromosomes.

### Effects of overweight on testicular function

**Figure 6:**
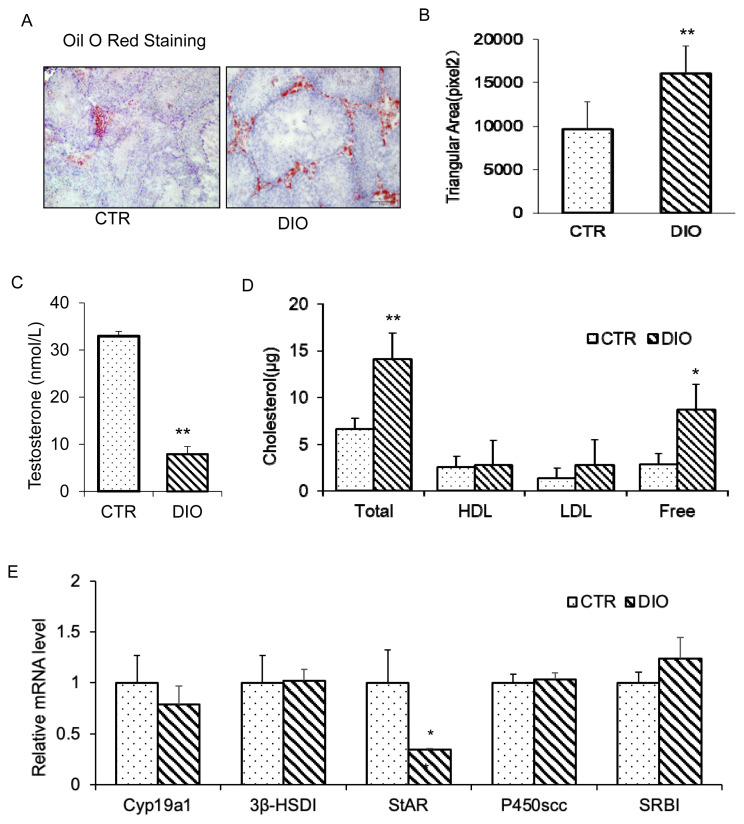
Fat deposition in the testis and the effects of fat deposition on the synthesis of testosterone. (A) Oil red staining of frozen sections of testicular tissue in the control group and DIO group. (B) Oil red coloring area comparison in the two groups. (C) Comparison of testicular testosterone levels. (D) Different types of cholesterol levels in mouse testes. (E) Expression of key genes in testosterone synthesis. DIO, diet-induced overweight. * p<0.05, ** p<0.01.

The testicles are essential male reproductive organs, and their proper function is crucial for male reproductive health. However, being overweight can negatively impact testicular function due to fat deposition in or around the testis. To investigate this further, we conducted oil red staining, which revealed that lipid droplets were mainly stored in or around the Leydig cells (Figure 6A). The staining also showed a significant difference in the oil red coloring area between the DIO and CTR groups (p<0.01) (Figure 6B). Moreover, we tested the testosterone content using an ELISA kit and found that the DIO group had significantly lower testosterone secretion than the CTR group (Figure 6C). Further analysis showed that the differences in cholesterol and testosterone material were primarily found in the free cholesterol (Figure 6D). Testosterone synthesis requires the synergistic action of multiple enzymes, and we found that the key enzyme steroidogenic acute regulatory protein (StAR) was significantly decreased in the overweight group (Figure 6E). Based on these results, the reduction in testosterone synthesis is a complex physiological activity. Fat deposition affects testosterone production by affecting the expression of related enzymes, such as StAR, which is crucial for testosterone synthesis.

## DISCUSSION

The present study was to investigate the impact of testicular fat deposition on animal reproduction and the underlying mechanisms. The study found that despite similar food intake, the DIO mice had a significant increase in body weight but not a decrease in offspring numbers. The hypothalamus produces GnRH, which triggers the pituitary gland to release FSH and LH. These hormones, known as gonadotropins, then travel to the testes where they stimulate Sertoli and Leydig cells involved in Spermatogenesis [25]. Fat accumulation is often associated with the hypothalamic-pituitary-gonadal axis, which inhibits GnRH levels and decreases FSH and LH release. Our research found that DIO mice exhibit lower levels of FSH, which is in line with previous studies. Therefore, the lower FSH level has a negative effect on testicular functions. However, based on our findings, there is a resemblance in LH levels among both groups, but it is not possible to attribute the low levels of testosterone in DIO mice solely to the lack of LH stimulation. Sperm quality was found to be diminished in DIO mice, as evidenced by lower sperm density and motility. Using immunofluorescence microscopy, we determined that fat accumulation in the testes had a direct impact on the meiosis process, specifically the exchange of homologous chromosomes. In addition, overweight mice had increased fat deposition in the testes, which led to decreased expression of enzymes involved in testosterone synthesis, ultimately affecting the production and secretion of this hormone by Leydig cells in the testes. Previous studies have shown that obesity escalates testicular fat accumulation and weight, while testicular cytology reduces sperm cell count [26]. Furthermore, there is an observed rise in Sertoli cell numbers and a decline in Leydig cell numbers. Because the function of Sertoli cells and Leydig cells is the primary manifestation of testicular function, testicular fat deposition affects testicular function. Another study examined testicular fat accumulation in 30 individuals, revealing a significant correlation between decreased testicular fat and improvements in both sperm count and motility. This study also supports our research results [27]. These results indicate that fat accumulation in the testes causes dysfunction in testicular cells, resulting in reduced testosterone synthesis, and ultimately impacting sperm formation.

We first investigate the link between overweight and reproductive ability by studying fat accumulation in mice. We found that fat accumulation leads to decreased sperm motility and density, which is consistent with previous studies [28,29]. In fact, under the stress of being overweight, testicular cells are prone to an inflammatory state. Therefore, regulating the mechanisms involved in lipid metabolism can effectively prevent overweight-related hypogonadism. Although our study confirmed that fat accumulation led to reduced sperm motility and density, there was no significant difference in the number of offspring in overweight male mice. Other studies also support this finding such as the research by Tortoriello [30] and his colleagues who found that male mice did not have reduced fertility even when consuming high-fat foods. Jedrzejczak et al [31] also emphasized that semen quality is a poor predictor of fertility, and the factors that cause low semen quality are not necessarily factors that lead to low fertility. The process of establishing a model of overweight in rats found that a high-fat, high-energy diet triggered testicular development problems. Other studies have also shown that long-term fat accumulation causes a decrease in semen quality in mice [32].

Meiosis is an important process in spermatogenesis [19, 33], that reduces the number of chromosomes in biological cells by half. During meiosis, non-sister chromatids of homologous chromosomes exchange genetic information, known as gene recombination, which diversifies the genetics of gametes and increases the adaptability of offspring to their environment. Therefore, meiosis is not only to ensure the stability of the number of chromosomes of biological species but also serves as a mechanism for species to continuously adapt to changes in the environment. Various proteins are involved in the process of chromosome cross-exchange, such as MLH1, a recombinant protein that plays a crucial role in determining the crossover process of chromosomes. Our results showed that abnormal expression of MLH1 leads to meiotic arrest, resulting in a reduced number of mature sperm.

The process of spermatogenesis and maturation depends on the testosterone. Our study found that testosterone contents in the DIO group was significantly lower than in the CTR group. Cholesterol is a key component in the synthesis of testosterone [7]. Early have shown that cholesterol is present in both the interstitial tissue and seminiferous tubules of the testes, but the specific mechanisms for the transport of cholesterol between cells in the testes are not well understood [11]. Therefore, we analyzed the cholesterol content of testicular tissue and found that the main difference between the DIO and CTR groups was in the levels of free cholesterol, not HDL or LDL.

Testosterone synthesis depends on the availability of cholesterol and the enzymes involved in the synthesis process [34]. Previous studies have confirmed that significant enzymes in testosterone synthesis are age-related [35]. Instead, the effect of age on testosterone synthesis was excluded by choosing same-aged mice as the study subjects. Cholesterol available to Cholesterol side-chain cleavage enzyme (P450scc) is the rate-limiting step in testosterone synthesis on the mitochondrial inner membrane [36]. Thus, we suggested that the metabolism of cholesterol and the transport in mitochondria involved in this process may hinder testosterone synthesis. The enzyme 3β-Hydroxysteroid dehydrogenase (3β-HSD), which is highly expressed in the gonads, plays a crucial role in converting fewer active substances into more active ones, and thus affects testicular interstitial cell function [36]. The analysis of the above vital metabolic enzymes revealed no significant difference in the expression of 3β-HSD and P450scc in the DIO group. While the expression level of StAR was significantly decreased, which may be the main reason for the decrease in testosterone secretion [37].

## CONCLUSION

Our finding suggests that fat accumulation in the testes causes testicular cell dysfunction, which affects testosterone hormone synthesis and ultimately affects sperm formation.

## Figures and Tables

**Table 1:** Composition of high-fat diets

Ingredients	High-fat diet (g/kg)	Kcal	Normal diet (g/kg)	Kcal
Casein	200	800	140	560
Corn oil	50	440	50	440
Cocoa buffer	70	224	0	0
Coconut oil	30	60	0	0
Lard	50	450	0	0
Cholesterol	5	150	0	0
Corn starch	190	760	190	760
Sucrose	300	1,200	300	1,200
Cellulose	50	0	50	0
Mineral mix	35	0	35	0
Vitamin mix	10	40	10	40
Methionine	3	0	3	0
Choline	2	0	2	0

**Table 2:** List of primary antibodies used in immunocytochemistry experiment

Target	Host	Company	Catalog number
Anti-VASA	Rabbit polyclonal antibody	Abcam	ab209710
Anti-MLH1	Mouse monoclonal antibody	BD Pharmingen	551091
Anti-SYCP3	Rabbit polyclonal antibody	Abcam	ab15093
Anti-γH2AX	Mouse monoclonal antibody	Millipore	05–636
Anti-SYCP1	Rabbit polyclonal antibody	Novus Biologicals	NB300-228

**Table 3:** Primers used for real time polymerase chain reaction

Genes	Sequences of primers
*Cyp19a1*	F:5′- CCCTGGTCTTGTTCGAATGGT -3′ R:5′- AATGCTGCTTGATGGACTCCA -3′
*3β-HSD*	F:5′- CTCAGTTCTTAGGCTTCAGCAATTAC -3′ R:5′- CCAAAGGCAAGATATGATTTAGGA -3′
*P450scc*	F:5′- CCAGTGTCCCCATGCTCAAC -3′ R:5′- TGCATGGTCCTTCCAGGTCT -3′
*StAR*	F:5′- CCGGAGCAGAGTGGTGTCA -3′ R:5′- CAGTGGATGAAGCACCATGC -3′
*SRBI*	F:5′- GCCCATCATCATCTGCCAACT -3′ R:5′- TCCTGGGAGCCCTTTTTACT -3′
*GAPDH*	F:5′- AGAACATCATCCCTGCATCCA -3′ R:5′- CCGTTCAGCTCTGGGATGAC -3′

*Cyp19a1*, cytochrome P450 19a1; *3β-HSD*, 3β-hydroxysteroid dehydrogenase; *StAR*, steroidogenic acute regulatory protein; *SRBI*, scavenger receptor class B type 1; *GAPDH*, glyceraldehyde-3-phosphate dehydrogenase.
